# A mutation in the intron splice acceptor site of a *GA3ox* gene confers dwarf architecture in watermelon (*Citrulluslanatus* L.)

**DOI:** 10.1038/s41598-020-71861-7

**Published:** 2020-09-10

**Authors:** Yuyan Sun, Huiqing Zhang, Min Fan, Yanjun He, Pingan Guo

**Affiliations:** Institute of Vegetables, Zhejiang Academy of Agricultural Sciences, Hangzhou, 310021 China

**Keywords:** Agricultural genetics, Plant molecular biology

## Abstract

Dwarf architecture is an important trait associated with plant yield, lodging resistance and labor cost. Here, we aimed to identify a gene causing dwarfism in watermelon. The ‘w106’ (dwarf) and ‘Charleston Gray’ (vine) were used as parents to construct F_1_ and F_2_ progeny. Dwarf architecture of ‘w106’ was mainly caused by longitudinal cell length reduction and was controlled by a single recessive gene. Whole-genome sequencing of two parents and two bulk DNAs of F_2_ population localized this gene to a 2.63-Mb region on chromosome 9; this was further narrowed to a 541-kb region. Within this region, Cla015407, encoding a gibberellin 3β-hydroxylase (GA3ox), was the candidate gene. Cla015407 had a SNP mutation (G → A) in the splice acceptor site of the intron, leading to altered splicing event and generating two splicing isoforms in dwarf plants. One splicing isoform retained the intron sequences, while the other had a 13-bp deletion in the second exon of *GA3ox* transcript, both resulting in truncated proteins and loss of the functional Fe2OG dioxygenase domain in dwarf plants. RNA-Seq analysis indicated that expression of Cla015407 and other GA biosynthetic and metabolic genes were mostly up-regulated in the shoots of dwarf plants compared with vine plants in F_2_ population. Measurement of endogenous GA levels indicated that bioactive GA_4_ was significantly decreased in the shoots of dwarf plants. Moreover, the dwarf phenotype can be rescued by exogenous applications of GA_3_ or GA_4+7_, with the latter having a more distinct effect than the former. Subcellular localization analyses of GA3ox proteins from two parents revealed their subcellular targeting in nucleus and cytosol. Here, a *GA3ox* gene controlling dwarf architecture was identified, and loss function of *GA3ox* leads to GA_4_ reduction and dwarfism phenotype in watermelon.

## Introduction

Plant height is an important agronomic trait that affects the quality and yield of plants^1^. The cultivation of dwarf crops has been widely applied in wheat^1^, rice^2^, maize^3^, pea^4^ and peach^5^. At present, most vine watermelon (*Citrullus* *lanatus* L.) varieties are planted for commercial production. With the rapid spread of watermelon protection areas, it is a good opportunity for the selection and cultivation of dwarf watermelon. Dwarf watermelon have short shoots, and are easily cultivated and managed, allowing for an increase in planting density, which can lead to increased yields and financial benefits.

There are various mechanisms leading to plant dwarfism. Changes in biosynthesis or perception of plant hormones, such as gibberellin (GA)^6^, brassinosteroid^7^, auxin^8^ and strigolactone^9^ can cause plant dwarfism. Additionally, abnormal expression of transcription factors, such as HD-Zip II^10^, WOX^11^, AP2^12^ and GRAS^13^ can also lead to dwarf architecture in plants.

GAs is a kind of diterpenoid carboxylic acids that widely exist in plants, including the functional active molecules GA_1_, GA_3_, GA_4_ and GA_7_, as well as the non-active molecules GA_9_, GA_19_, GA_20_, GA_29_ and GA_51_^14,15^. GAs have a variety of biological functions, including promoting the stem elongation^14,15^. In plants, GA biosynthesis and metabolism are catalyzed by six key enzymes, ent-copalyl diphosphate synthase (CPS), ent-kaurene synthase (KS), ent-kaurene oxidase (KO), ent-kaurenoic acid oxidase (KAO), GA 20-oxidase (GA20ox) and GA 3-oxidase (GA3ox), and its deactivation is catalyzed by GA 2-oxidase (GA2ox)^14,15^. GAs could promote the stem elongation by stimulating the degradation of DELLA proteins^16^. Mutations in GA biosynthetic genes lead to dwarfism by reducing the endogenous GA levels, leading to DELLA protein accumulation, and ultimately limiting internode elongation in plants^5^.

Among the six enzymes, GA20ox and GA3ox catalyze the final two steps of GA biosynthetic pathway, and GA2ox catalyzes the GA metabolic pathway^17–19^. *GA20ox*, the “Green revolution gene” in rice, encodes a key enzyme that catalyzes the penultimate step of GA biosynthesis, converting GA_12_ to GA_9_, and GA_53_ to GA_20_^17^. Mutations in *GA20ox* genes conferring dwarf phenotypes have been reported in rice^20,21^, barley^22,23^ and *Arabidopsis*^24,25^. GA3ox catalyzes the final step of the GA biosynthetic pathway, converting GA_20_ to GA_1_, GA_9_ to GA_4_, and GA_5_ to GA_3_, which leads to the active molecules GAs^18^. Mutations of *GA3ox* genes have been identified in maize^18^, rice^26^, *Arabidopsis*^27^ and *Medicago* *sativa*^28^, and they are causing the dwarf phenotypes in these plants. GA2ox catalyzes the deactivation of bioactive GAs or its precursors to inactive forms through 2β-hydroxylation reaction; thus, plays a direct role in the determination of bioactive GAs content^19^. The *GA2ox* genes leading to dwarf phenotypes have been reported in wheat^6^, switchgrass^29^, rice^30^ and tomato^31^.

Watermelon belongs to the *Cucurbitaceae* and is a diploid species with a chromosomal number of 2*n* = 2 × 11 and a genome size of 425 Mb^32^. Approximately 97 million tons of watermelon are produced worldwide each year, with China being the largest producer. Watermelon dwarf mutants have been identified and analyzed in previous studies. Dwarf mutants in watermelon are controlled by *dw-1*^33^, *dw-1*^*s*^^34^, *dw-2*^35^, *dw-3*^36^, *dsh*^37^, *Cldf*^38^, *dw*^39^ and *Cldw-1*^40^, and the dwarf traits were controlled by respective single recessive genes^37–40^. At present, the *dsh* gene has been identified through whole-genome sequencing of two bulk DNAs, and Cla010726 (*GA20ox*) was predicted to be the candidate gene^37^. In addition, a SNP mutation of *GA3ox*^38,39^ and a SNP deletion in an ABC transporter gene^40^ also lead to dwarfism phenotypes in watermelon.

In recent years, the combination of a bulked segregant analysis (BSA) and next-generation sequencing technology (BSA-Seq) has been widely applied to identify candidate genes controlling important agronomic traits in watermelon, such as dwarf phenotype^37–40^, lobed leaves^41^, yellow skin^42^, fruit shape^43^, fruit pigment accumulation^44^ and anthracnose resistance^45^. In this study, we investigated the inheritance of watermelon dwarf genes in the F_2_ population of ‘w106’ (dwarf) × ‘Charleston Gray’ (CG; vine), which indicated that the dwarf phenotype was controlled by a single recessive gene. The candidate gene, Cla015407 (*GA3ox*), was obtained through BSA-Seq and mapping analysis. A single nucleotide polymorphism (SNP) mutation (G → A) occurred in the splice acceptor site of the intron in Cla015407, which lead to altered splicing, resulting in two splicing isoforms in dwarf plants. This point mutation leads to loss function of *GA3ox* and GA_4_ level reduction in dwarf plants. This study identify a *GA3ox* gene controlling dwarf architecture in watermelon and will aid in revealing the molecular mechanism of plant height in future.

## Materials and methods

### Plant materials

Two watermelon parental lines, ‘w106’ (dwarf) and ‘CG’ (vine), were used as the female and male parents, respectively. The F_1_ plants were generated by crossing ‘w106’ and ‘CG’, and self-pollinated to produce the F_2_ progeny. The 15 plants of each parental line, 15 F_1_ plants and 98 F_2_ plants were used for genetic analysis and BSA-Seq. For fine mapping, the dwarf individuals of a larger F_2_ population were used. The cross and self-pollination were carried out at the Haining Base of Zhejiang Academy of Agricultural Sciences. The plants used for the genetic analysis, BSA-Seq and fine mapping were grown and evaluated in a greenhouse at Zhejiang Academy of Agricultural Sciences.

### Analysis of shoot sections

Shoots of dwarf and vine plants were fixed with 50% FAA for 24 h at 4 °C. Subsequently, these samples were dehydrated in a graded ethanol series, infiltrated with xylene and embedded in paraffin. Sections were sliced using an ultramicrotome and stained with safranin and fast green, and finally, observed under an optical microscope.

### Bulk DNA construction and Illumina sequencing

DNAs were extracted using the CTAB method from leaves of both parental lines and F_2_ plants for BSA-Seq. Two bulk DNA samples, dwarf bulk (D-bulk) and vine bulk (V-bulk), were constructed by mixing equal amounts of DNAs from 25 dwarf plants and 25 vine plants from the F_2_ population, respectively. Paired-end DNA libraries were prepared according to the manufacturer’s instructions (Truseq Library Construction; Illumina, San Diego, CA, USA). First, genomic DNA was sheared into 350-bp fragments using a Covaris S220 sonicator (Woburn, MA, USA). Second, ends of the gDNA fragments were repaired, and 3′ ends were adenylated. Then, the size distributions and concentrations of the libraries were determined using an Agilent 2100 Bioanalyzer (Agilent Technologies, Waldbronn, Germany) and quantified by real-time PCR. Finally, DNA libraries were sequenced using an Illumina HiSeq X at Genepioneer (Nanjing, Jiangsu, China) according to the manufacturer’s instructions for paired-end 150-bp reads.

The short reads from D-bulk and V-bulk were aligned to the ‘97103’ reference genome^32^ using BWA software^46^. Alignment files were converted to SAM/BAM files using SAM tools^47^. SNPs and Insertion/deletion polymorphisms (InDels) were also assessed.

### Gene location association analysis

All samples underwent variant calling using the Unified Genotyper function of the GATK program^48^. The SNPs and InDels were filtered using the Variant Filtration parameter of GATK. ANNOVAR, which is an efficient software tool, was used to annotate the SNPs or InDels based on the GFF3 files for the reference genome^49^. The homozygous SNPs/InDels between two parental lines were extracted from the vcf files.

A SNP index was used to indicate the proportion of reads harboring SNPs that differed from reference sequences^50^. An Euclidean distance (ED) value was calculated by comparing SNPs across the two bulk DNAs as follows: SNP-index_alt_ = N_alt_/(N_alt_ + N_ref_), Δ(SNP-index_alt_) = SNP-index_alt_ (V-bulk) −SNP-index_alt_ (D-bulk), SNP-index_ref_ = N_ref_/(N_alt_ + Nref), Δ(SNP − index_ref_) = SNP − index_ref_ (V-bulk) − SNP-index_ref_ (D-bulk) and ED = [Δ(SNP-index_ref_)^2^  + Δ (SNP-index_alt_)^2^]^1/2^^51^. Using these formulae, we assessed whether the measured values fell within the following ranges, − 1 ≤ Δ(SNP-index) ≤ 1 and 0 ≤ ED ≤ 1.414^51,52^. The greater of the ED value, the closer of the object site^52^. The Δ(InDel-index) and EDs of InDel sites were calculated using the InDel-index^53^ as described above for calculating for SNP regions. Using a 1-kb sliding window, an average SNP/InDel-index was calculated over a 1-Mb interval.

### Mapping of the candidate gene

To minimize the genetic interval and verify the accuracy of BSA-Seq, 161 simple sequence repeat (SSR) markers within the BSA-Seq region were developed based on the whole-genome sequencing of the two parental lines. These newly developed SSR markers were first screened for polymorphisms between the two bulk DNAs, then the polymorphic SSRs were used to screen for recombinants in the dwarf individuals of the F_2_ population.

The PCR was carried out in a total volume of 15 μL containing 7.5 μL 2 × TSINGKE Master Mix (Tsingke, Beijing, China), 0.5 μL of each primer (10 μM), 2 μL genomic DNA (~ 50 ng/μL) and 4.5 μL sterilized ddH_2_O. All the amplifications were performed on a Mastercycler nexus GSX1 (Eppendorf, Germany) under the following conditions: 95 °C for 5 min; 33 cycles of 30 s at 95 °C, 45 s at 55 °C and 45 s at 72 °C, followed by a final extension step at 72 °C for 10 min. The amplified products were separated on 8.0% non-denatured polyacrylamide gel with electrophoresis at 150 V constant power for 1 h. After fixation in 10% ethanol + 0.5% glacial acetic acid for 10 min, the silver staining in 0.2% AgNO_3_ for 12 min was performed. Samples were then rinsed in distilled water for 1 min and 1.5% NaOH + 0.4% formaldehyde for 6 min. The band pattern analysis was performed under a GL-800 Compact White Light Transmissometer (Kylin-Bell, Haimen, Jiangsu, China).

### Cloning and sequence analysis of candidate gene

Total DNAs and RNAs were extracted from leaves of two parental plants using CTAB and TRIzol Reagent (Invitrogen, Carlsbad, CA, USA), respectively. First-strand cDNA was synthesized using a FastKing RT Kit (with gDNase) (Tiangen Biotech, Beijing, China). The PCR was carried out in a total volume of 25 μL containing 12.5 μL 2 × PCR buffer for KOD FX (Toyobo, Osaka, Japan), 0.5 μL KOD FX (1.0 U/μL) (Toyobo), 5.0 μL dNTPs (2 mM) (Toyobo), 0.5 μL of each primer (10 μM) (F: 5′-ATGGGAAGCATCAAAATAACCG-3′; R: 5′-TTAACCTACTTTAACCTGGCTG-3′), 2.0 μL cDNA (50 ng/μL) and 4.0 μL sterilized ddH_2_O. Amplifications of candidate genes were performed under the following conditions: 95 °C for 5 min; 33 cycles of 30 s at 95 °C, 45 s at 55 °C and 45 s at 72 °C, followed by a final extension step at 72 °C for 10 min. Amplification products were analyzed on 1.5% agarose gel and sent for sequencing.

### Transcriptome sequencing of dwarf and vine plants

Transcriptome sequencing (RNA-Seq) was performed to analyze the expression of candidate genes and reveal the related pathways involved in dwarf architecture. Total RNAs were extracted from shoots of dwarf and vine plants in the F_2_ population using TRIzol Reagent (Invitrogen, Carlsbad, CA, USA). Approximately 10 µg total RNA was subjected to Poly(A) mRNA isolation using poly-T oligo-attached magnetic beads (Invitrogen, Carlsbad, CA, USA). Following purification, the mRNA was fragmented into small pieces and the cleaved RNA fragments were reverse-transcribed to create the final cDNA library. The average insert size for the paired-end libraries was 300 bp (± 50 bp). Then, the paired-end sequencing was performed on an Illumina HiSeq 4000 platform at Genepioneer (Nanjing, Jiangsu, China) following the vendor’s recommended protocol. Triplicates of each sample were carried out for Illumina sequencing.

Gene expression was assessed using the fragments per kilobase of transcript per million fragments mapped (FPKM) method^54^. The differentially expressed genes (DEGs) were determined using the criterion |log_2_(Fold Change)|≥ 1 and FDR < 0.05. The corresponding functions were revealed using the KEGG Automatic Annotation Server^55^.

### Measurement of endogenous GA levels using internal standards

At the fourth-true leave stage, the shoots of vine and dwarf plants in two parents and F_2_ population grown under the same condition were harvested. The endogenous levels of 18 kinds of GAs involved in GA biosynthetic and metabolic pathway (GA_1_, GA_3_, GA_4_, GA_5_, GA_6_, GA_7_, GA_8_, GA_9_, GA_12_, GA_15_, GA_19_, GA_20_, GA_24_, GA_29_, GA_34_, GA_44_, GA_51_ and GA_53_) were measured using 2H2-GA internal standards coupled with UPLC-MS/MS analyses at LC Sciences (Hangzhou, China). For the measurement, shoots from three seedlings of vine and dwarf plants were mixed and ground into fine powder in liquid nitrogen, respectively; weighed ~ 100 mg sample, added 1.0 ng/g corresponding 2H2-GA internal standards and 1 mL methanol/water (80/20, v/v) extracting solution at 4 °C for overnight, then centrifuged at 10,000×*g* for 20 min at 4 °C; the supernatant was taken and absorbed through the C18/SAX solid-phase extraction column; 2 mL methanol/water (20/80, v/v) was used to clean the SAX extraction column, and 3 mL ACN/FA (99/1, v/v) was used to desorb the target acid plant hormones retained on the SAX extraction column; the desorption solution was blow-dried with constant nitrogen flow at 40 °C and redissolved in 100 μL water, and 10 μL FA was added to the 100 μL solution and extracted twice with ether; the extracted organic phase was combined and blow-dried by nitrogen at room temperature, then dissolved in 100 μL ACN; adding 10 μL × 20 μmol/mL TEA and 10 μL × 20 μmol/mL BTA to the ACN solution, swirl for 35 min at room temperature and then blow-dried with nitrogen; redissolved the solution in 200 μL H_2_O/ACN (90/10, v/v) for subsequent UPLC-MS/MS analyses. The UPLC-MS/MS analyses were performed on Thermo Scientific Ultimate 3000-Thermo Scientific TSQ Quantiva. Three biological replicates were conducted for each measurement, and endogenous levels of GAs (ng/g fresh weight) were determined by means of three UPLC-MS/MS detection results. T-test was conducted for statistical analysis using SAS 8.0. The GAs with |log2Fold(FC)| ≥ 1 and statistical significance (*p* value < 0.05) were considered as significant difference.

### Exogenous treatments with GA_3_ or GA_4+7_

In addition, the dwarf parental lines were employed to assess responses to exogenous application of GAs. The GA_3_ (Ryon, Shanghai, China) and GA_4+7_ (Ryon, Shanghai, China) are independently dissolved in a small amount of ethanol and then diluted with sterilized ddH_2_0 to the final concentration of 500 μM. The seedlings were sprayed independently with 500 μM GA_3_ or GA_4+7_ for four times at 3-day intervals. The control seedlings were sprayed with sterilized ddH_2_0. Three seedling were used for each treatment. The phenotypes of seedlings were analyzed and photographed 3-days after the last GA treatment.

### Subcellular localization analyses of GA3ox protein

The CDS sequences of Cla015407 from ‘CG’ and ‘w106’ were amplified using gene-specific primer (F: 5′-CGGGATCCCGATGGGAAGCATCAAAATAAC-3′; R: 5′-GCTCTAGAGCTTAACCTACTTTAACCTGGCTG-3′) and cloned into the pFGC-eGFP plasmid via the BamH I and Xba I restriction sites. These recombinant plasmids were transformed into *Agrobacteriumt* *tumefaciens* GV3101 and transiently expressed in tobacco leaf cells. Images were acquired at 48 h using a Leica DMLE camera (Leica, Wetzlar, Germany).

### Ethical standards

The authors declare that this study complies with the current laws of the countries in which the experiments were performed.

## Results

### Phenotypic and genetic analyses

**Figure 1:**
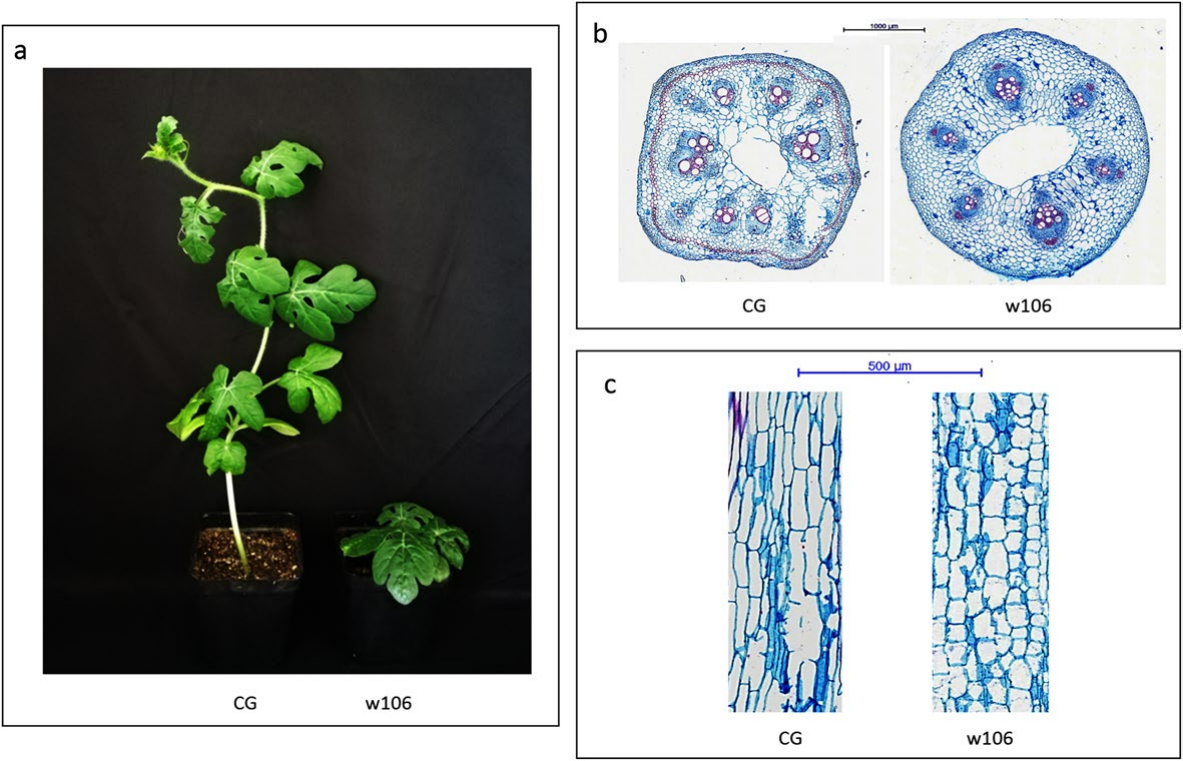
Phenotypic characterization and morphological analysis of two parental lines used in present study. (**a**) Phenotypic characterization of the two parental lines. Shoot length are reduced in ‘w106’ (right) as compared to ‘CG’ (left). (**b**) Transverse sections of shoots in two parental lines. (**c**) Longitudinal sections of shoots in two parental lines. The ‘w106’ (right) showed reduced longitudinal cell length compared with ‘CG’ (left).

The height of ‘w106’ was significant shorter than that of ‘CG’ (Fig. 1a). Therefore, we conducted a microscopic observation of shoots of the ‘CG’ and ‘w106’ plants using paraffin sectioning. The cell sizes in transverse sections were not obviously different between the ‘CG’ and ‘w106’ plants (Fig. 1b). However, the cell lengths in longitudinal sections were obviously shorter in ‘w106’ than in ‘CG’ plants (Fig. 1c). Thus, the defective cell elongation appears to be the main cause for the reduced shoots and dwarf architecture in watermelon.

To assess the inheritance of the dwarf trait in watermelon, crosses were made between ‘w106’ and ‘CG’. All the F_1_ plants showed the vine phenotype. Among the 98 F_2_ progeny, 72 individuals showed vine phenotype and 26 individuals showed dwarf phenotype, with the Chi-square test (χ^2^) confirming the segregation ratio to be 3:1 (Table 1). These results indicated that the dwarf trait in ‘w106’ was controlled by a single recessive gene, designated as the *short* *internode* (*Si*) locus.

**Table 1:** Genetic analysis of the dwarf trait in watermelon.

Plants	Number of total plants	Number of vine plants	Number of dwarf plants	Χ^2^_3:1_	χ^2^_0.05_
‘w106’ (female; dwarf)	15	0	15		
‘CG’ (male; vine)	15	15	0		
F_1_	15	15	0		
F_2_	98	72	26	0.683	3.841

χ^2^_3:1_ < χ^2^_0.05_ = 3.841, indicating that dwarf trait in ‘w106’ was controlled by a single recessive gene.

### *Si* gene located on chromosome 9 (0.80–3.43 Mb) using BSA-seq

**Figure 2:**
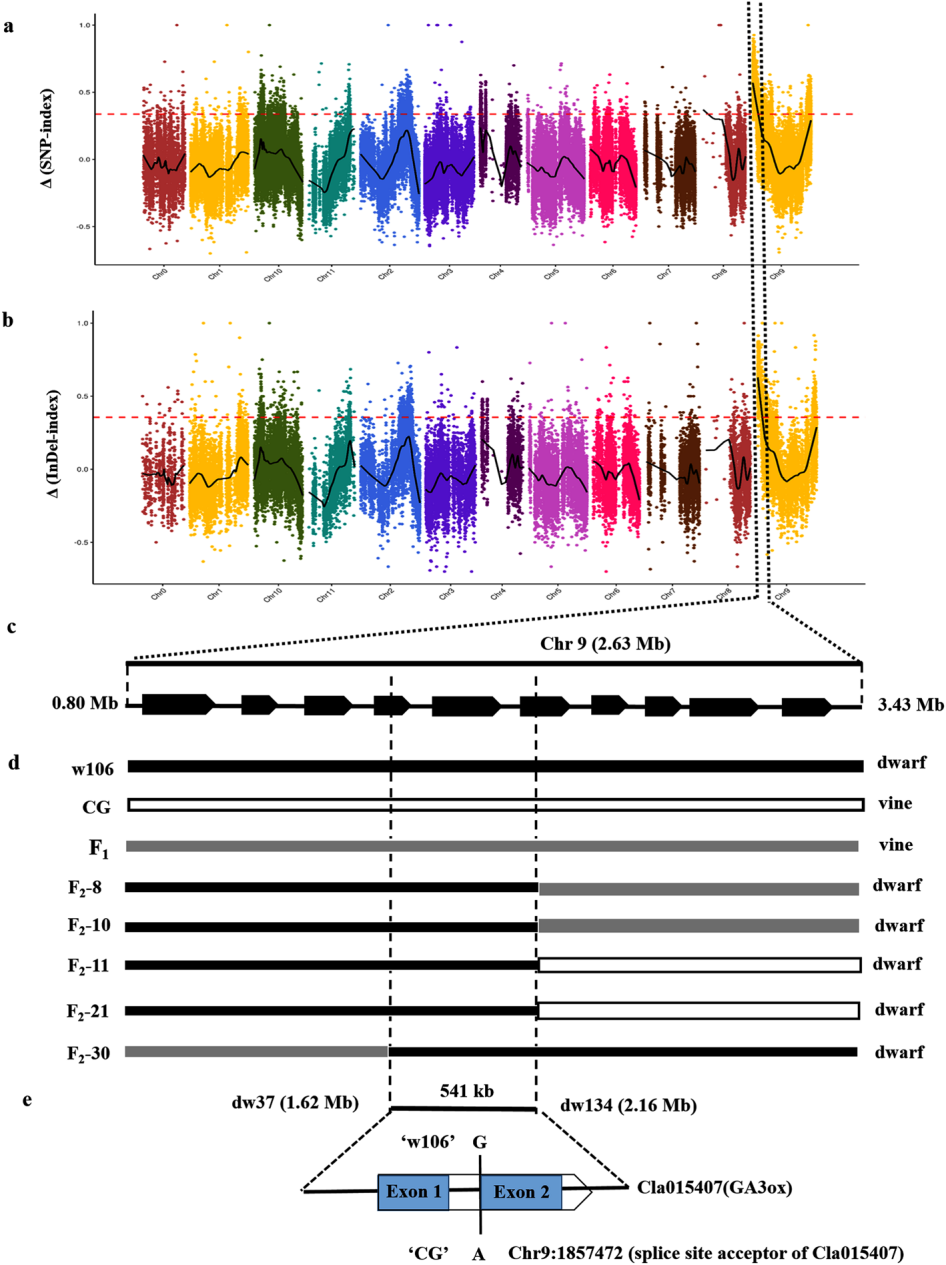
Genetic mapping and prediction of the *Si* locus in watermelon by BSA-Seq and mapping analysis. (**a**) Δ(SNP-index) graph of BSA-Seq analysis. (**b**) Δ(InDel-index) graph of BSA-Seq analysis. (**c**) Locus at the interval of 0.80–3.43 Mb on chromosome 9 was identified to control watermelon dwarf trait by combining the Δ(SNP-index) and Δ(InDel-index) of BSA-Seq analysis. The *x*-axis represents the position of watermelon chromosomes. The *y*-axis represents the value of Δ-index for SNPs and InDels. The scatter in the figure indicates that the value of Δ-index is calculated. The black curve represents the fitting value of Δ-index and the pink dotted line represents a threshold line with an snpnum smooth fitting value of 99%. (**d**) Screening of recombinants in F_2_ progeny delimited the location of *Si* in an interval of 541-kb by two SSR markers dw37 and dw134. (**e**) Within the 541-kb interval, a SNP change occurring in the splice site acceptor for the intron of Cla015407, which encodes the GA3ox protein.

We sequenced the genomes of the two parental lines and two bulk DNAs using the Illumina HiSeq™ PE150 platform. The high-throughput sequencing results obtained 50.30, 61.89, 65.48 and 70.68 Mb clean reads for female parent, male parent, D-bulk and V-bulk, respectively (Table 2). A total of 33.65 Gb clean data were generated for the two parental lines, and 40.84 Gb clean data were generated for the two DNA bulks, with approximately 42–57 × sequencing depth and more than 99.00% 5 × coverage per sample (Table 2). Data were aligned to the reference genome of watermelon ‘97103’ (https://cucurbitgenomics.org/organism/1), and 160,957 SNPs and 55,055 InDels, at a minimum of 5 reads, were identified between D-bulk and V-bulk. Each identified SNP or InDel was used to compute an SNP/InDel-index. The graph for Δ(SNP/InDel-index) was plotted and computed against the genome positions by combining SNP/InDel-index of D-bulk and V-bulk (Fig. 2a,b). At the 99% significance level, the Δ(SNP-index) and Δ(InDel-index) values located the region on chromosome 9 (0.72–3.93 Mb) and chromosome 9 (0.80–3.43 Mb), respectively (Fig. 2a,b; Supplementary Table S2). The results indicated that a candidate gene controlling the dwarf trait in ‘w106’ was located in the 0.80–3.43 Mb region of chromosome 9 (Fig. 2c).

**Table 2:** Summary of sequencing data for two parental lines and two DNA bulks in F_2_ population.

Samples	Number of reads (M)	Clean reads (M)	Clean data (G)	Q20 (%)	Q30 (%)	Average depth	Coverage (≥ 5 ×) (%)
‘w106’ (female)	102.32	50.30	15.09	96.64	91.00	42.87	99.44
‘CG’ (male)	126.70	61.89	18.56	96.78	91.31	50.93	99.45
D-bulk	133.59	65.48	19.64	96.78	91.31	55.09	99.48
V-bulk	144.67	70.68	21.20	96.99	91.79	57.95	99.48

### Further mapping analysis narrowed *Si* to a 541-kb interval and a candidate gene was predicted

To further narrow down the location of the *Si* locus detected by BSA-Seq, we selected 161 SSR markers from chromosome 9 (0.80–3.43 Mb) based on resequencing data of the two parental lines. All these SSR markers were first screened for polymorphisms between the two bulk DNA samples, and then, 16 polymorphic markers were applied to screen recombinants of the dwarf individuals in the F_2_ population. Finally, two flanking markers, dw37 (Chr9:1620039) and dw134 (Chr9:2161629), obtaining one and four recombinants, respectively, placed the *Si* locus in a 541-kb region (Fig. 2d). Additionally, no recombinant was obtained using the marker dw128 (Chr9:1835342), indicating that the target gene neighbored dw128. All the polymorphic SSR markers used in this study and the obtained recombinants are listed in Supplementary Table S3.

According to the watermelon genome annotation, 66 putative genes (Cla015361–Cla015427) were detected within the 541-kb interval (Supplementary Table S4). Within this region, three SNPs and one InDel were identified among the two parental lines and two bulk DNAs according to the whole-genome sequencing data (Table 3). Among these four variations, two SNP variations (Chr9:1620753 and Chr9:1621230) occurred in the intergenic region of the genome and an InDel (Chr9:1996536) occurred in the upstream of Cla015387 (WD43). One SNP occurred in Chr9:1857472 (from ‘G’ in ‘CG’ to ‘A’ in ‘w106’), locating at the splice-site acceptor in the intron of Cla015407 (Fig. 2e; Table 3). Cla015407 encodes GA3ox, which is involved in the GA biosynthetic pathway. Additionally, the genome location of Cla015407 (Chr9:1856847–1858103) neighbored the SSR marker dw128 (Chr9:1835342), which did not identify any recombinants and was near the target gene. Therefore, Cla015407 was predicted to be the candidate gene conferring the dwarf architecture of ‘w106’.

**Table 3:** SNP and InDel variations within the region on Chr9 (1.62–2.16 Mb).

Variation types	Positions	‘w106’ (dwarf)	‘CG’ (vine)	D-bulk	V-bulk	Effect	Codon change /distance	Gene ID	Nr_annotation
SNP	Chr9:1620753	A, A	G, G	A, A	A, G	Intergenic	–	–	
SNP	Chr9:1621230	T, T	C, C	T, T	T, C	Intergenic	–	–	
SNP	Chr9:1857472	A, A	G, G	A, A	G, A	Splice site acceptor	Exon_2	Cla015407	Gibberellin 3β-hydroxylase
InDel	Chr9:1996536	AT, AT	ATAC,ATAC	AT, ATAC	ATAC, ATAC	Upstream	286	Cla015387	WD repeat-containing protein 43

*Intergenic* DNA sequences located between genes (no transcript), *splice* *site* *acceptor* splice donor mutation (within 2 bp before exon), *Upstream* upstream gene region (within 5-kb), *Codon* *change/distance* coding changes (old_codon/new_codon) or mutation distance to transcript (upstream and downstream region of gene).

### Sequences analyses of the candidate gene

**Figure 3:**
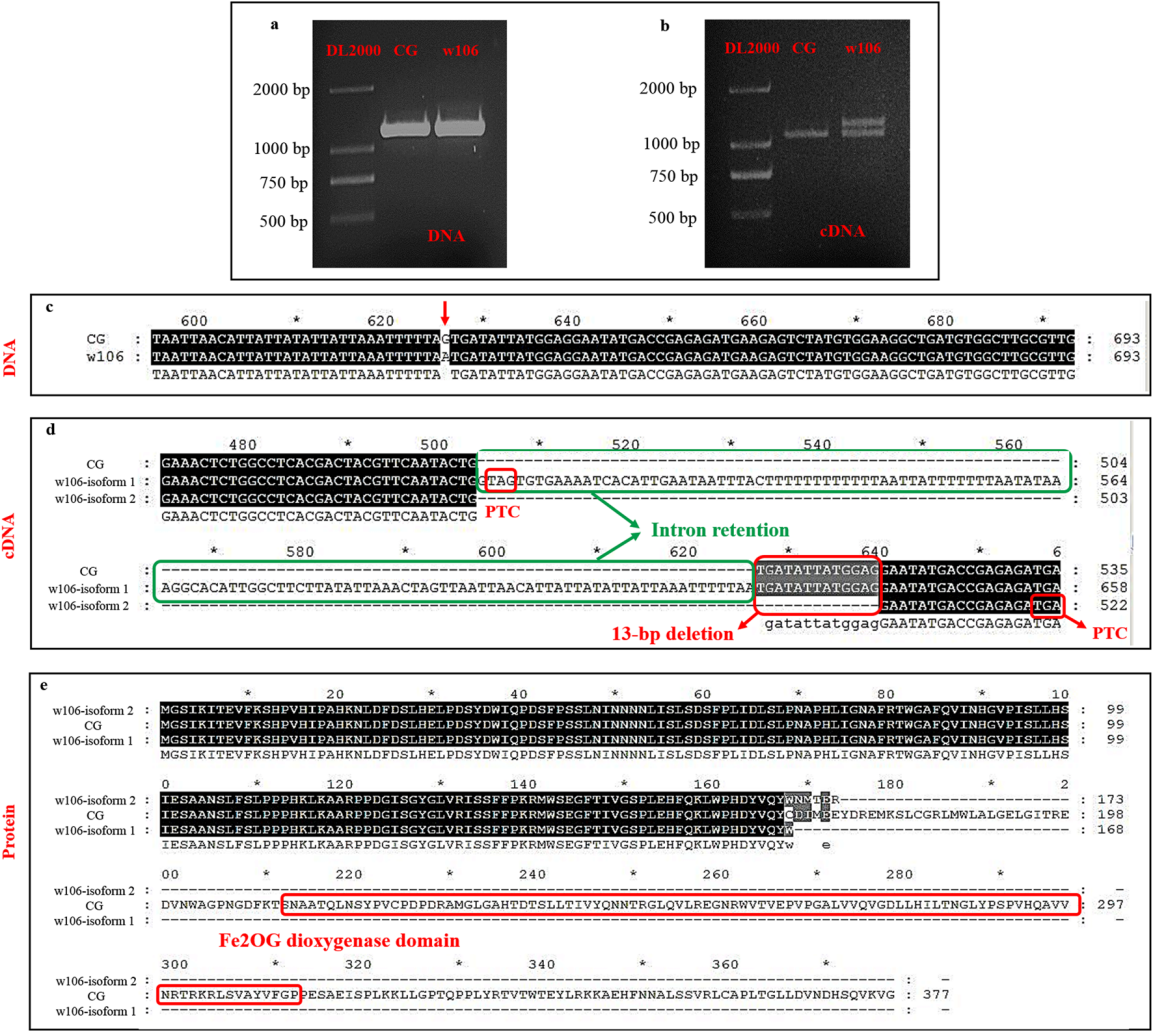
Sequence analysis of Cla015407 from two parental lines. (**a**) Amplifications of Cla015407 in two parental lines on DNA level. (**b**) Amplifications of Cla015407 in two parental lines on cDNA level. (**c**) Sequencing results verified the SNP (G → A) in ‘CG’ and ‘w106’ on DNA level at 626th nucleotide. (**d**) Sequencing results of two splicing isoforms on cDNA level in ‘w106’ indicated that the full-length isoform retained the intron sequence and the truncated isoform had 13-bp deletion at the second exon compared with the CDS in ‘CG’. (**e**) Amino acids prediction of the two splicing isoforms indicted the loss of Fe2OG dioxygenase domain in ‘w106’ compared with ‘CG’.

To verify the sequences of Cla015407 at the DNA and mRNA levels, we cloned the DNA and coding sequence (CDS) of Cla015407 from both parental lines. Fragments of 1,257 bp were amplified at the DNA level from both parental lines (Fig. 3a), and the sequencing analysis further verified the G → A variation at the 626th nucleotide of Cla015407 (Fig. 3c).

At the cDNA level, a fragment was amplified from vine parent ‘CG’ and two fragments were amplified from dwarf parent ‘w106’, which indicated that this SNP mutation lead to altered splicing, generating two splicing isoforms in the dwarf plants (Fig. 3b). Sequence analyses of the splicing isoforms in ‘w106’ revealed that the full-length isoform (isoform 1) retained the intron sequences and contained the premature termination codon ‘TAG’ at the 505–507th nucleotides (Fig. 3d). Additionally, the truncated isoform (isoform 2) had a 13-bp deletion in the second exon compared with the CDS of ‘CG’ and contained the premature termination codon ‘TGA’ at the 520–522th nucleotides (Fig. 3d).

The proteins encoded by the transcripts of Cl015407 in vine and dwarf parents were also predicted. The transcript of Cl015407 in vine parent ‘CG’ encodes a protein with 377 aa (Fig. 3e). However, the full-length isoform (isoform 1) in the dwarf parent ‘w106’ contains an unspliced intron, introducing a stop codon (TAG) just after the splice donor site, thus translation of this full-length transcript is prematurely terminated and produces a protein with 168 aa (Fig. 3e). Moreover, the truncated isoform (isoform 2) has a 13-bp deletion in the second exon of Cla015407 in dwarf parent ‘w106’ and contains a premature termination codon ‘TAG’ at 520–522th nucleotides, leading to frame shift and premature termination, and resulted in a truncated protein with 173 aa residues (Fig. 3e). In summary, the two transcripts of Cl015407 from the dwarf plants resulted in truncated proteins and lost the functional Fe2OG dioxygenase domain.

### DEGs identification between dwarf and vine plants and their KEGG pathway enrichment analyses

**Figure 4:**
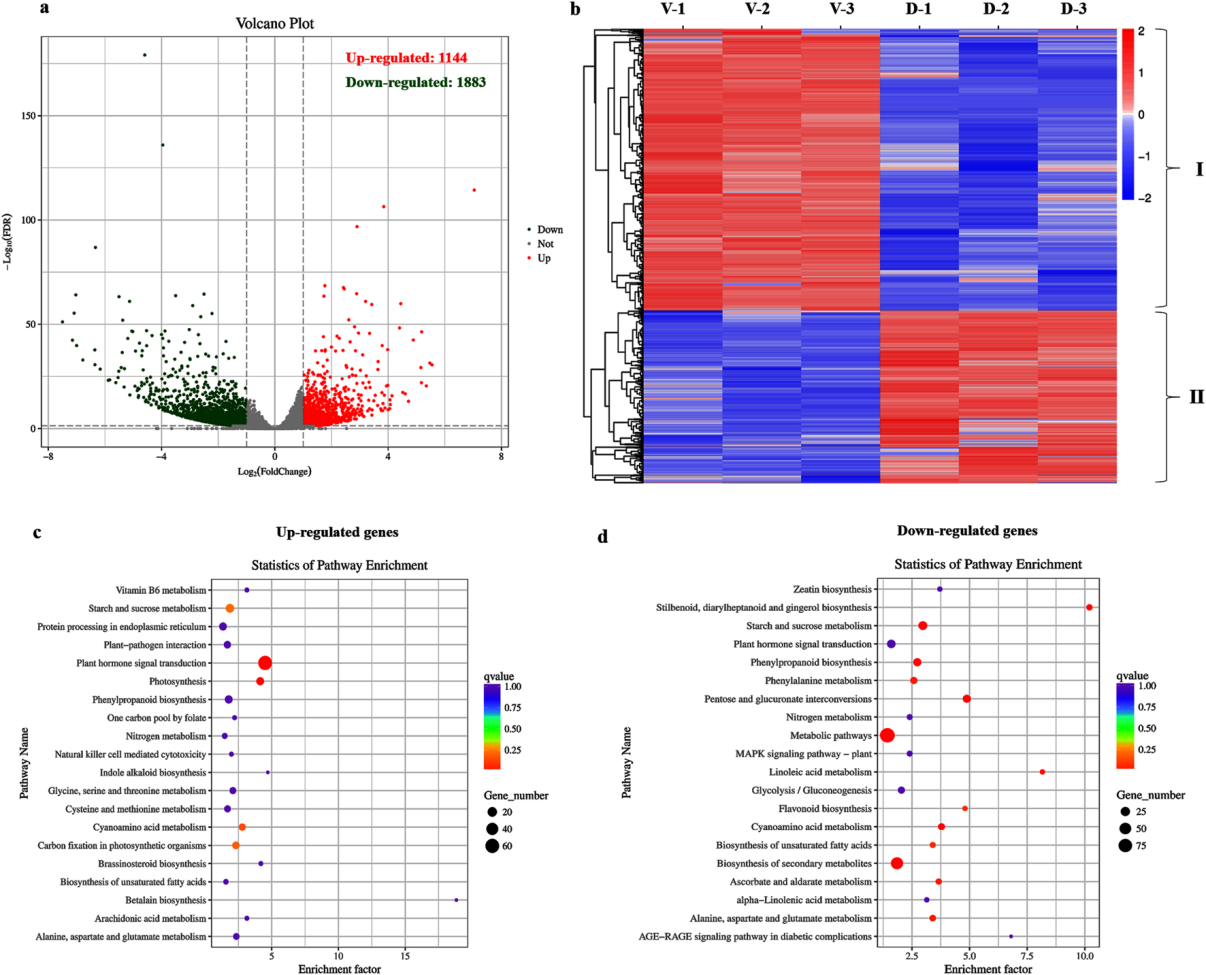
Volcano plot and heatmap of DEGs and their functional analysis. (**a**) Volcano plot of DEGs for vine plants and dwarf plants in F_2_ population. (**b**) Heatmap of DEGs for vine plants and dwarf plants in F_2_ population. The expression of DEGs were indicated by log2(FPKM + 0.001). (**c**) The KEGG pathways enriched for the up-regulated genes in dwarf plants compared with vine plants. (**d**) The KEGG pathways enriched for the down-regulated genes in dwarf plants compared with vine plants. The size of each circle represents the number of significantly DEGs enriched in the corresponding pathway. The enrichment factor was calculated using the number of enriched genes divided by the total number of background genes in the corresponding pathway. The *q* value was calculated using the Benjamini–Hochberg correction. A pathway with *q* < 0.05 is considered significantly overrepresented.

Transcriptome analyses of the shoots for dwarf and vine plants in the F_2_ population were carried out to reveal the expression patterns of the candidate gene and GA biosynthetic and metabolic genes. A total of 3,027 genes showed differential expression, with 1,144 up-regulated and 1,883 down-regulated in dwarf plants compared with vine plants (Fig. 4a). In addition, the heatmap generating by Cluster 3.0 clearly divided these 3,017 DEGs into two Clusters (I and II) (Fig. 4b).

The KEGG pathway enrichment analyses were carried out for these DEGs (Fig. 4c,d). The up-regulated genes in dwarf plants were significantly enriched in KEGG pathways of ‘plant hormone signal transduction’ and ‘photosynthesis’ (Fig. 4c). Moreover, the down-regulated genes in dwarf plants were significantly enriched in KEGG pathways of ‘pentose and glucuronate interconversions’, ‘biosynthesis of secondary metabolites’, ‘stilbenoid, diarylheptanoid and gingerol biosynthesis’, ‘starch and sucrose metabolism’, ‘metabolic pathways’, ‘phenylpropanoid biosynthesis’, ‘cyanoamino acid metabolism’, ‘linoleic acid metabolism’, ‘alanine, aspartate and glutamate metabolism’, ‘ascorbate and aldarate metabolism’, and ‘phenylalanine metabolism' (Fig. 4d).

### Expression of GA biosynthetic and metabolic genes and phylogenetic analysis

As shown in Table 4, a total of 20 genes involved in GA biosynthesis and metabolism were detected as expressed at least one library, including one gene for CPS, one gene for KS, one gene for KO, two genes for KAO, six genes for GA20ox, two genes for GA3ox and seven genes for GA2ox. Most of these GA biosynthetic and metabolic genes in dwarf plants showed higher expression levels than those of vine plants. The expression of our candidate gene, Cla015407 (*GA3ox*), was significantly increased in dwarf plants. Moreover, one *KO* gene (Cla020710) and three *GA20ox* genes (Cla002362, Cla006227 and Cla008413) involved in GA biosynthesis, and two *GA2ox* gene (Cla015162 and Cla019586) involved in GA metabolism, were also significantly up-regulated in dwarf plants compared with those in vine plants.

**Table 4:** Expression of GA biosynthetic and metabolic genes by transcriptome analysis.

Gene	Annotation	FPKM						Up/down-regulation
		V-1	V-2	V-3	D-1	D-2	D-3	
Cla006048	Ent-copalyl diphosphate synthase (CPS)	4.76	10.14	5.55	7.06	6.49	8.06	Up
Cla005482	Ent-kaurene synthase 1 (KS)	9.53	8.75	8.23	11.12	11.21	13.53	Up
Cla020710	Ent-kaurene oxidase (KO)	12.92	13.62	10.71	28.40	24.53	29.08	Up (sig)
Cla006992	Ent-kaurenoic acid oxidase 1 (KAO)	14.56	24.91	31.02	23.16	16.90	20.05	Down
Cla021351	Ent-kaurenoic acid oxidase 1 (KAO)	1.66	1.41	1.27	1.72	1.20	1.42	-
Cla002362	Gibberellin 20-oxidase (GA20ox)	1.39	0.43	1.08	9.77	10.05	10.28	Up (sig)
Cla006227	Gibberellin 20-oxidase (GA20ox)	0.00	0.00	0.00	0.71	0.16	0.38	Up (sig)
Cla006941	Gibberellin 20-oxidase (GA20ox)	0.47	0.52	1.17	1.03	0.30	0.44	Down
Cla008413	Gibberellin 20-oxidase (GA20ox)	0.00	0.00	0.04	0.28	0.18	0.67	Up (sig)
Cla010726	Gibberellin 20-oxidase-like protein (GA20ox)	3.98	3.64	3.63	5.68	5.27	5.58	Up
Cla013892	Gibberellin 20-oxidase (GA20ox)	0.09	0.16	0.04	0.19	0.18	0.26	Up
Cla015407	Gibberellin 3-beta-hydroxylase (GA3ox)	4.56	3.86	4.13	11.86	12.36	10.67	Up (sig)
Cla022285	Gibberellin 3-beta-hydroxylase (GA3ox)	0.05	0.00	0.00	0.30	0.14	0.04	Up
Cla005259	Gibberellin 2-beta-dioxygenase 8 (GA2ox)	0.00	0.00	0.19	0.00	0.11	0.52	Up
Cla005397	Gibberellin 2-oxidase (GA2ox)	2.92	8.67	2.63	3.29	4.50	2.05	Down
Cla007482	Gibberellin 2-beta-dioxygenase 8 (GA2ox)	1.53	1.35	1.24	1.13	1.31	1.95	Up
Cla009774	Gibberellin 2-oxidase (GA2ox)	0.00	0.18	0.17	0.27	0.36	0.05	Up
Cla015162	Gibberellin 2-oxidase (GA2ox)	0.05	0.05	0.00	0.84	0.72	0.75	Up (sig)
Cla017338	Gibberellin 2-oxidase 2 (GA2ox)	0.00	0.00	0.05	0.62	0.03	0.20	Up
Cla019586	Gibberellin 2-oxidase (GA2ox)	0.32	1.53	0.87	6.71	3.51	9.32	Up (sig)

‘V’ indicates the vine plants in the F_2_ population; ‘D’ indicates the dwarf plants in the F_2_ population; ‘up/down’ indicates the up/down-regulation of genes in dwarf plants compared with the vine plants; ‘sig’ indicates the significantly up/down-regulated gene. The table listed the genes expressed at least a library and those GA biosynthetic and metabolic genes did not express in any library were not listed.

Additionally, phylogenetic analysis of these GA20ox, GA3ox and GA2ox proteins in watermelon were carried out with a few selected GA20ox, GA3ox and GA2ox families in *Arabidopsis* and divided them into three major subgroups (I, II and Ш) (Supplementary Fig. S1). Subgroup I contained five watermelon GA20ox proteins (Cla002362, Cla006227, Cla006941, Cla008413 and Cla013892) and two Arabidopsis GA20ox proteins (AT5G07200 and AT1G44090). Subgroup II contained five watermelon GA2ox proteins (Cla015162, Cla017338, Cla005397, Cla009774 and Cla019586) and two Arabidopsis GA2ox proteins (AT1G47990 and AT1G30040). Subgroup Ш contained two GA3ox proteins (Cla022285 and Cla015407), two GA2ox proteins (Cla005259 and Cla007482) from watermelon and two GA3ox proteins (AT4G21690 and AT1G15550) from Arabidopsis. However, Cla010726, encoding the gibberellin 20-oxidase-like protein, did not belong to any of these subgroups.

### Endogenous levels of GAs were changed in the dwarf plants

Except for GA_7_, the remaining 17 kinds of GAs were detected from the shoots of vine and dwarf plants in two parents and F_2_ population using the 2H2-GA internal standards coupled with UPLC-MS/MS analyses. Among these GAs, endogenous level of GA_3_ was significantly increased and GA_4_ was significantly decreased in dwarf plants of two parents and F_2_ population (Table 5). Additionally, endogenous levels of GA_9_ and GA_29_ were significantly increased in dwarf plants of F_2_ population. The results indicated the reduced level of bioactive GA_4_ might be the main cause for the dwarf phenotype in watermelon (Table 5).

**Table 5:** Measurement of endogenous levels (ng/g fresh weight) of GAs in dwarf and vine plants in two parents and F_2_ population using 2H2-GA internal standards coupled with UPLC-MS/MS analyses.

Types of GAs	CG, vine parent (± SD)	w106, dwarf parent (± SD)	Vine plants of F_2_ population (± SD)	Dwarf plants of F_2_ population (± SD)
GA_1_	0.0152 ± 0.0003	0.0210 ± 0.0016	0.0270 ± 0.0007	0.0230 ± 0.0004
GA_3_	n.d	0.0527 ± 0.0027* ↑	0.0268 ± 0.0010	0.1029 ± 0.0034* ↑
GA_4_	0.1074 ± 0.0026	0.0118 ± 0.0011* ↓	0.0900 ± 0.0064	0.0176 ± 0.0016* ↓
GA_5_	0.0223 ± 0.0020	0.0225 ± 0.0006	0.0216 ± 0.0007	0.0217 ± 0.0009
GA_6_	0.0081 ± 0.0002	0.0100 ± 0.0012	n.d	0.0084 ± 0.0014
GA_7_	n.q	n.q	n.d	n.q
GA_8_	0.0256 ± 0.0039	0.0275 ± 0.0018	0.0275 ± 0.0009	0.0325 ± 0.0028
GA_9_	0.0494 ± 0.0016	0.0452 ± 0.0016	0.0371 ± 0.0012	0.0992 ± 0.0046* ↑
GA_12_	0.0882 ± 0.0088	0.0516 ± 0.0044	0.0466 ± 0.0080	0.0362 ± 0.0014
GA_15_	n.d	0.0882 ± 0.0025	n.d	n.d
GA_19_	0.1935 ± 0.0135	0.3053 ± 0.0220	0.1906 ± 0.0208	0.1770 ± 0.0183
GA_20_	0.0394 ± 0.0081	0.0309 ± 0.0011	0.0329 ± 0.0034	0.0469 ± 0.0038
GA_24_	0.0800 ± 0.0013	0.0652 ± 0.0019	0.0633 ± 0.0021	0.0575 ± 0.0022
GA_29_	0.0607 ± 0.0015	0.1136 ± 0.0018	0.0549 ± 0.0005	0.2391 ± 0.0045* ↑
GA_34_	0.0364 ± 0.0023	n.d	n.d	n.q
GA_44_	0.1218 ± 0.0017	0.1695 ± 0.0158	0.1193 ± 0.0075	n.q
GA_51_	0.0187 ± 0.0016	0.0277 ± 0.0025	0.0239 ± 0.0024	0.0263 ± 0.0034
GA_53_	0.0093 ± 0.0005	0.0152 ± 0.0011	0.0058 ± 0.0008	0.0044 ± 0.0008

Mean values of three independent UPLC–MS/MS runs. *Means GA level between two parents and the dwarf and vine plants in F_2_ population are significantly different at *p* < 0.05 by T-test using SAS 8.0 and |log2Fold(FC) |≥ 1. SD means standard deviation. ‘n.d.’ means no decision and ‘n.q.’ means no quantitation.

**Figure 5:**
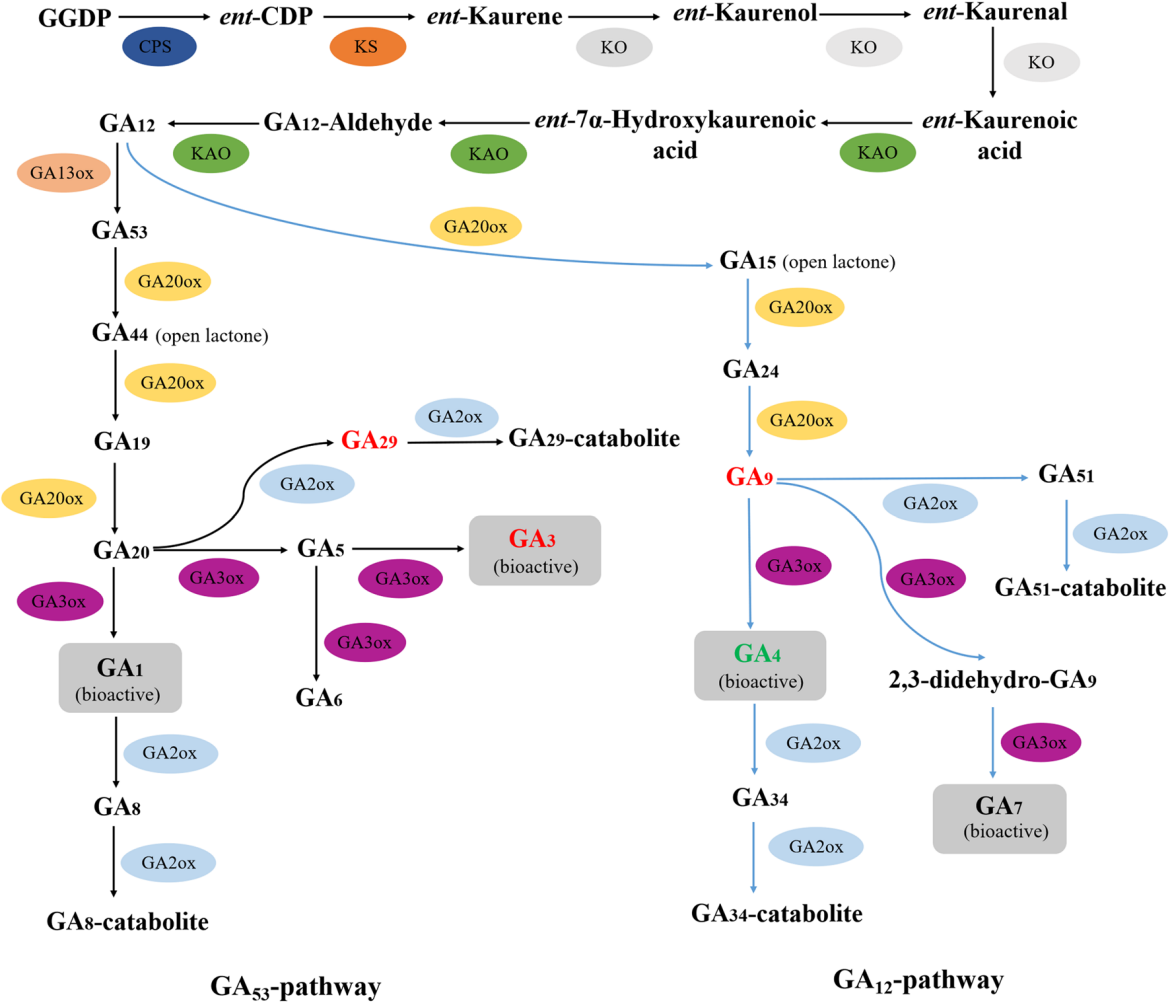
The predicted pathways of GA biosynthesis and metabolism in watermelon. The pathways were constructed according to the previous reports of GA biosynthetic and metabolic pathways in higher plants. Two major pathways, GA_53_-pathway and GA_12_-pathway were present in watermelon. The levels of GA_3_ and GA_29_ in GA_53_-pathway were increased in dwarf plants and marked with red color. The level of GA_9_ in GA_12_-pathway was increased and marked with red color, and level of GA_4_ in GA_12_-pathway was decreased in dwarf plants and marked with green color.

Moreover, we constructed the GA biosynthetic and metabolic pathway combining the endogenous levels of GAs and genes involved in this pathway (Fig. 5). Two major pathways, GA_53_-pathway (involving GA_53_, GA_44_, GA_19_, GA_20_, GA_1_, GA_8_, GA_29_, GA_5_, GA_3_ and GA_6_) and GA_12_-pathway (involving GA_12_, GA_15_, GA_24_, GA_9_, GA_4_, GA_34_, GA_51_ and GA_7_), were included. The GA_9_ and GA_4_ in the GA_12_-pathway showed different changes of endogenous levels between the vine and dwarf plants (Fig. 5; Table 5). Endogenous level of GA_9_ was increased in dwarf plants of F_2_ population, which might be due to the increased expression level of *GA20ox* genes (Cla002362, Cla006227 and Cla008413) in dwarf plants as indicated in Table 4. Endogenous level of bioactive GA_4_ was decreased in dwarf plants, suggesting the mutation of Cla015407 (*GA3ox*) impaired the biosynthesis of GA_4_ in GA_12_-pathway. Endogenous level of GA_29_ and GA_3_ in the GA_53_-pathway were increased in dwarf plants (Fig. 5; Table 5). The increased GA_29_ content might be attributed to the increased expression level of *GA2ox* genes (Cla015162 and Cla019586) in dwarf plants as indicated in Table 4. Endogenous level of bioactive GA_3_ was increased in dwarf plants, which might be due to the up-regulation of another *GA3ox* paralogue, Cla022285, as a result of feedback of reduced GA_4_ content caused by the mutation of Cla015407 (*GA3ox*) (Table 4).

### Exogenous GA applications can rescue the dwarf phenotype

**Figure 6:**
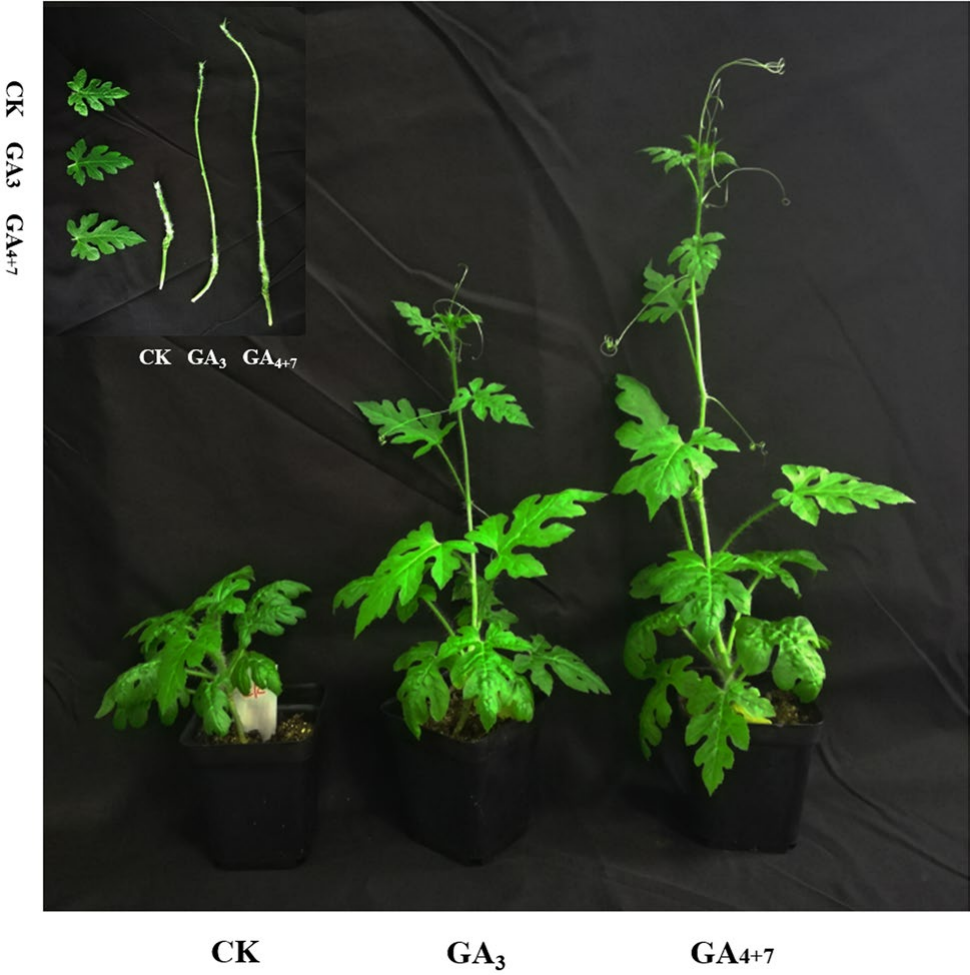
Dwarf architecture could be rescued after GA_3_ or GA_4+7_ treatment.

We investigated the responses of dwarf plants to 500 µM GA_3_ and 500 µM GA_4+7_ applications and found that the independent applications of GA_3_ or GA_4+7_ could both rescue the dwarf phenotype in watermelon (Fig. 6). Moreover, GA_4+7_ treatments had a more distinct effect than GA_3_ treatments, resulting in greater plant height (Fig. 6). These observations further verified that the *Si* gene is a GA biosynthetic gene.

### Subcellular localization of GA3ox proteins

**Figure 7:**
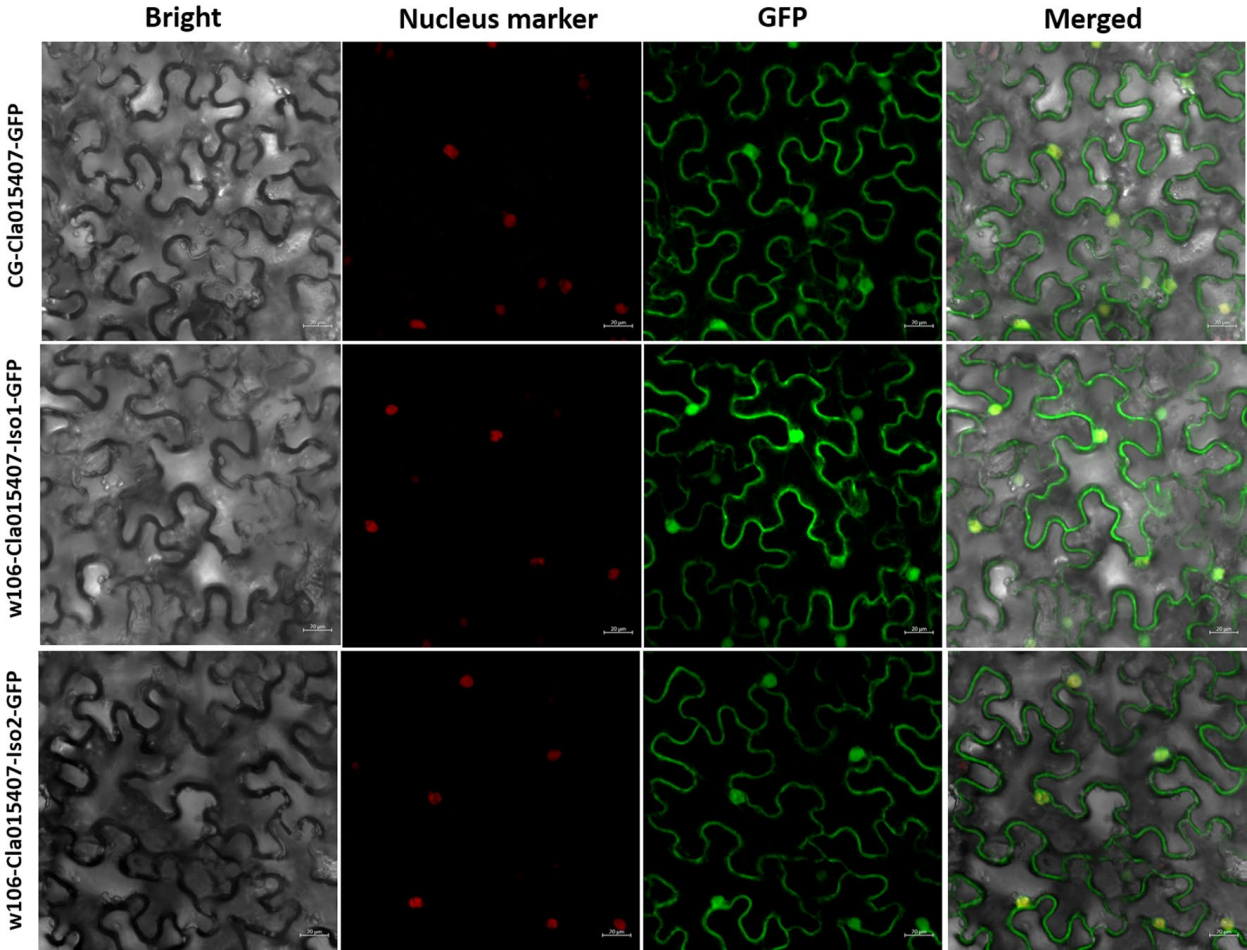
Subcellular localization of GA3ox proteins from two parental lines. Green fluorescent protein (GFP)-fusion proteins were transiently expressed in tobacco leaf epidermal cells. After 48 h of incubation, GFP signal was detected with a fluorescence microscope. Bright-field, nucleus marker, fluorescence, and merged images of CG-Cla015407-GFP, w106-Cla015407-Iso1-GFP, and w106-Cla015407-Iso2-GFP were shown.

The subcellular localization of GA3ox proteins from ‘CG’ and ‘w106’, namely CG-Cla015407, w106-Cla015407-Iso1, and w106-Cla015407-Iso2, were analyzed by transient expression of the green fluorescent protein (GFP) fusion proteins in tobacco leaf epidermal cells. As shown in Fig. 7, all of the three GA3ox proteins were localized in the nucleus and cytosol.

## Discussion

Plant height, as the key component of plant architecture, has been associated with both natural beauty and yield performance. Total cell number and cell size, resulting from cell division and expansion, determine the size of plant organs^56^. For example, the average cell size of the cucumber dwarf mutant *scp2* was significantly smaller than that of wild type plants^57^. The cucumber *Csdw* mutant showed dwarfing phenotype and decreased internode length due to the reduced cell division in main stem^58^. The rice *stemless* *dwarf* *1* (*std1*) mutant showed severe dwarfing phenotype, abnormal cell morphology and reduced cell division rate^59^. Further analyses revealed that a large number of cells were blocked in the S and G2/M phases, with the presence of many multinucleate cells^59^. In present study, microscopic observations of stem transverse and longitudinal sections revealed that the reduced plant height could be mainly attributed to the shorter longitudinal cell lengths (Fig. 1b,c).

Genetic mapping and identification of dwarfism genes have occurred in cucurbits, such as *scp-2*^57^, *Csdw*^58^, *cp*^60^ and *scp-1*^61^ in cucumber; *mdw1*^62^ in melon; and *qCmB2*^63^ in pumpkin. In watermelon, the genes of *dsh*^37^, *Cldf*^38^, *dw*^39^ and *Cldw-1*^40^, conferring dwarf phenotypes, were studied and identified. NGS-assisted BSA is an effective method to identify target genes controlling the dwarf traits by genotyping the bulked DNA samples having distinct or opposite extreme phenotypes in plants^58^. In this study, we employed the BSA-Seq to identify the candidate gene controlling the dwarf trait in watermelon and delimited the region to 0.80–3.43 Mb on chromosome 9 (Fig. 2a–c). A further mapping analysis finally located this gene in a 541-kb interval (Fig. 2d), with Cla015407 (*GA3ox*) being the candidate gene.

GA3ox catalyzes the last step of bioactive GA synthesis, which converts GA_20_ to GA_1_, GA_5_ to GA_3_, and GA_9_ to GA_4_^18^. Mutations in *GA3ox* genes, such as *dwarf1* (*d1*) from maize^18^, *dwarf18* (*d18*) from rice^26^, *GA4* from Arabidopsis^27^, *Msdwf1* from *Medicago* *sativa*^28^, and *le* from pea^64^, resulted in dwarfism. For example, the coding sequences of *GA3ox2* isolated from *d18* alleles were analyzed in rice. In the strong allele, *d18-Id18*^*h*^, the deletion of a guanine base at 750 altered the reading frame, and in the weak allele, *d18-dy*, the 9-bp deletion deleted three amino acids^26^. The dwarf mutant *Msdwf1* had an amino acid change in a conserved position of the *GA3ox* gene compared with the wild type in *Medicago* *sativa*^28^. Sequence alignment revealed a G-to-A transition conferring an alanine-to-threonine substitution at residue 229 in the *le* gene product in pea^64^. The same locus of *GA3ox*, Cla015407, conferring the dwarf phenotypes in watermelon were concurrently identified in this study and those of Wei et al^38^ and Gebremeskel et al^39^. Additionally, the SNP mutation (G → A) at position 626 of DNA sequence, locating at the splice acceptor site of intron, was simultaneously detected in our study and those of Wei et al^38^ and Gebremeskel et al^39^. Similar with previous reports, the SNP mutation leading to 13-bp deletion in the second exon of *GA3ox* transcript, was also observed in our dwarf parent ‘w106’. Moreover, another *GA3ox* transcript caused by this point mutation, which retained the intron sequence, was identified in our dwarf parent ‘w106’. However, this full-length isoform was not present in the dwarf parents ‘N21’ and ‘Duan125’ of Wei et al^38^ and Gebremeskel et al^39^.

In the present study, the candidate gene, Cla015407 (*GA3ox*), significantly increased the expression level in dwarf plants compared with the vine plants (Table 4). Additionally, one *KO* gene (Cla020710), three *GA20ox* genes (Cla002362, Cla006227 and Cla008413) and two *GA2ox* genes (Cla015162 and Cla019586) were also significantly up-regulated in dwarf plants (Table 4). The increased expression of GA related genes in this study were partially consistent with Wei et al^38^, in which the *GA3ox* (Cla015407), *CPS* (Cla006048), *KAO* (Cla021351, Cla006992 and Cla016164), and *GA20ox* (Cla002362, Cla006227 and Cla010726) were significantly up-regulated in dwarf line ‘N21’. Different from our results, the expression of *GA3ox* (Cla015407) in the dwarf parent ‘Duan125’ was significantly reduced according to the report of Gebremeskel et al.^39^. The increase in expression of GA related genes in our study might be due to the feedback of low levels of bioactive GA_4_ in dwarf plants as showing in Table 5. This is a well-known phenomenon whereby mutations or chemical intervention in GA biosynthesis or GA perception result in increases in *GA20ox* and *GA3ox* in a homeostatic mechanism^14^. Moreover, two transcripts of *GA3ox*, the intron retention isoform and 13-bp deletion isoform, were generated in the dwarf parent ‘w106’ in our study. Introns are often added to increase expression, although the mechanism through introns stimulate gene expression in plants remains unclear^65^. For instance, inserting the first intron from the *UBQ10* gene into intronless genes markedly increased the latter’s mRNA accumulation to over 150-fold in *Arabidopsis*^65^. Therefore, the up-regulated *GA3ox* gene might also be attributed to altered splicing event in dwarf plants.

Endogenous levels of GAs were measured using 2H2-GA internal standards and UPLC-MS/MS and we find that GA_3_, GA_9_ and GA_29_ were significantly increased and GA_4_ was significantly decreased in dwarf plants (Fig. 5; Table 5). The results were different from a previous study of Gebremeskel et al^39^, in which significantly lower GA_3_ content was obtained in the dwarf parent ‘Duan125’ of watermelon. The increased content of GA_9_ in GA_12_-pathway might be resulted from the up-regulation of *GA20ox* genes (Cla002362, Cla006227 and Cla010726) in dwarf plants, and the reduced content of bioactive GA_4_ indicated that loss function of *GA3ox* (Cla015407) impaired the GA_4_ biosynthesis in GA_12_-pathway. Furthermore, GA_51_ content in GA_12_-pathway was increased in dwarf plants, however, not to a significant level. The results indicate that mutation of *GA3ox* (Cla015407) promotes the biosynthesis of GA_51_ and inhibits the biosynthesis of GA_4_ from GA_9_ in GA_12_-pathway. Moreover, endogenous level of GA_29_ in GA_53_-pathway was increased, which might be attributed to the increased expression level of *GA2ox* genes (Cla015162 and Cla019586) in dwarf plants. Additionally, endogenous level of bioactive GA_3_ in GA_53_-pathway was increased in dwarf plants, which aroused our interest. We speculated that the reduction in GA_4_ level in GA_12_-pathway resulted in increased expression, through release of feedback, of another *GA3ox* paralogue, Cla022285, with the ability to produce GA_3_ in GA_53_-pathway (Table 4; Fig. 5). In rice, two *GA3ox* genes, *OsGA3ox1* and *OsGA3ox2*, have the activity for converting GA_20_ to GA_1_, GA_5_ to GA_3_, GA_44_ to GA_38_, and GA_9_ to GA_4_^26^. Additionally, the maize *dwarf-1* gene (putative GA 3β-hydroxylase) controls the three biosynthetic steps: GA_20_ to GA_1_, GA_20_ to GA_5_, and GA_5_ to GA_3_^66^. However, there is no evidence that Cucurbits contains such an GA3ox enzyme currently^67^. In cucumber, four GA 3-oxidases (CsGA3ox1, -2, -3, and -4) were identified and all these GA 3-oxidases oxidized the C_19_-GA GA_9_ to GA_4_ as the only product^67^. In this study, the presences of GA_1_, GA_5_, GA_6_, GA_3_ and GA_4_ indicate that the GA3ox proteins have the activity for catalyzing GA_20_ to GA_1_, GA_20_ to GA_5_, GA_5_ to GA_6_, GA_5_ to GA_3_, and GA_9_ to GA_4_, and the absence of GA_7_ suggests that the GA3ox proteins do not have 2,3-desaturation activity in watermelon.

The rescue of the dwarf phenotype has been reported in GA-deficient mutants in plants. For instance, the application of GA_3_ could partially rescue the dwarf phenotype of the cucumber mutant *Csdw*^58^. In watermelon, the dwarf phenotypes of *Cldf* and *dw* could be rescued by exogenous GA_3_ application^38,39^. In the present study, exogenous applications of GA_3_ or GA_4+7_ could rescue the height of dwarf plants, with the latter have a more distinct affect than the former (Fig. 6). The results further confirmed the *Si* gene is a GA biosynthetic gene and the dwarf phenotype might be attributed to the reduced GA_4_ level.

## Accession of sequencing data

Whole-genome sequencing data from this study can be accessed at sequence read archive (SRA) database from NCBI (https://www.ncbi.nlm.nih.gov/sra) with accession number SRR8893166 (female parent ‘w106’), SRR8893167 (male parent ‘Charleston Gray’), SRR8893168 (dwarf bulk) and SRR8893169 (vine bulk).

## Supplementary information

Supplementary file1.

Supplementary Table 1.

Supplementary Table 2.

Supplementary Table 3.

Supplementary Table 4.
