# Supplementary material for: A mutation in the intron splice acceptor site of a GA3ox gene confers dwarf architecture in watermelon (Citrulluslanatus L.)
Source: Sci Rep. 2020 Sep 10;10:14915. doi: 10.1038/s41598-020-71861-7 (PMC7483442; doi:10.1038/s41598-020-71861-7)
Supplement: Supplementary file 1 — Supplementary file1. [file 41598_2020_71861_MOESM1_ESM.pdf]

Supplementary information

Manuscript title: A mutation in the intron splice acceptor site of a GA3ox gene confers dwarf architecture in watermelon (*Citrullus lanatus* L.)

Abstract: Dwarf architecture is an important trait associated with plant yield and quality. Here, we aimed to identify a gene causing dwarfism in watermelon. The 'w106' (dwarf) and 'Charleston Gray' (vine) were used as parents to construct F_1 and F_2 progeny. Dwarf architecture of 'w106' was mainly caused by longitudinal cell length reduction and was controlled by a single recessive gene. Whole-genome sequencing of two parents and two bulk DNAs of F_2 population localized this gene to a 2.63-Mb region on chromosome 9; this was further narrowed to a 541-kb region. Within this region, Cla015407, encoding a gibberellin 3β-hydroxylase (GA3ox), was the candidate gene. Cla015407 had a SNP mutation (G→A) in the splice site acceptor of the intron, leading to altered splicing event and generating two splicing isoforms in dwarf plants. One splicing isoform retained the intron sequences, while the other had a 13-bp deletion in the second exon of *GA3ox* transcript, both resulting in truncated proteins and loss of the functional Fe2OG dioxygenase domain in dwarf plants. RNA-Seq analysis indicated that expression of Cla015407 and other GA biosynthetic and metabolic genes were mostly up-regulated in the shoots of dwarf plants compared with vine plants in F_2 population. Measurement of endogenous GA levels in shoots of dwarf and vine plants indicated that bioactive GA_4 was significantly decreased in the dwarf plants. Moreover, the dwarf phenotype can be rescued by exogenous applications of GA_3 or GA_{4+7}, with the latter having a more distinct effect than the former. Subcellular localization analyses of GA3ox proteins from two parents revealed their subcellular targeting in nucleus and cytosol. Here, a *GA3ox* gene controlling dwarf architecture was identified in watermelon, and the altered splicing events may play crucial roles in controlling plant height.

Keywords: watermelon; dwarf; BSA-Seq; GA3ox; transcriptome; UPLC-MS/MS

Contributing author details

Author lists: Yuyan Sun, Huiqing Zhang, Min Fan*, Yanjun He & Pingan Guo

First author: Dr. Yuyan Sun

Institute of Vegetables, Zhejiang Academy of Agricultural Sciences, Hangzhou 310021, China

Tel: +86 0571 8698 2260

E-mail: syy1111@126.com

Second author: Miss Huiqing Zhang

Institute of Vegetables, Zhejiang Academy of Agricultural Sciences, Hangzhou 310021, China

Tel: +86 0571 8640 8128

E-mail: Zhq111925@126.com
***Corresponding author:** Prof. Min Fan
Institute of Vegetables, Zhejiang Academy of Agricultural Sciences, Hangzhou 310021, China
Tel: +86 0571 8641 6057
E-mail address: fanminfm@sina.com
Forth author: Dr. Yanjun He
Institute of Vegetables, Zhejiang Academy of Agricultural Sciences, Hangzhou 310021, China
Tel: +86 0571 8640 8128
E-mail: hyj1009@163.com
Fifth author: Dr. Pingan Guo
Institute of Vegetables, Zhejiang Academy of Agricultural Sciences, Hangzhou 310021, China
Tel: +86 0571 8640 8128
E-mail: xc-guo12@hotmail.com

Competing Interests information

The authors declare no competing interests.

Supplementary figures

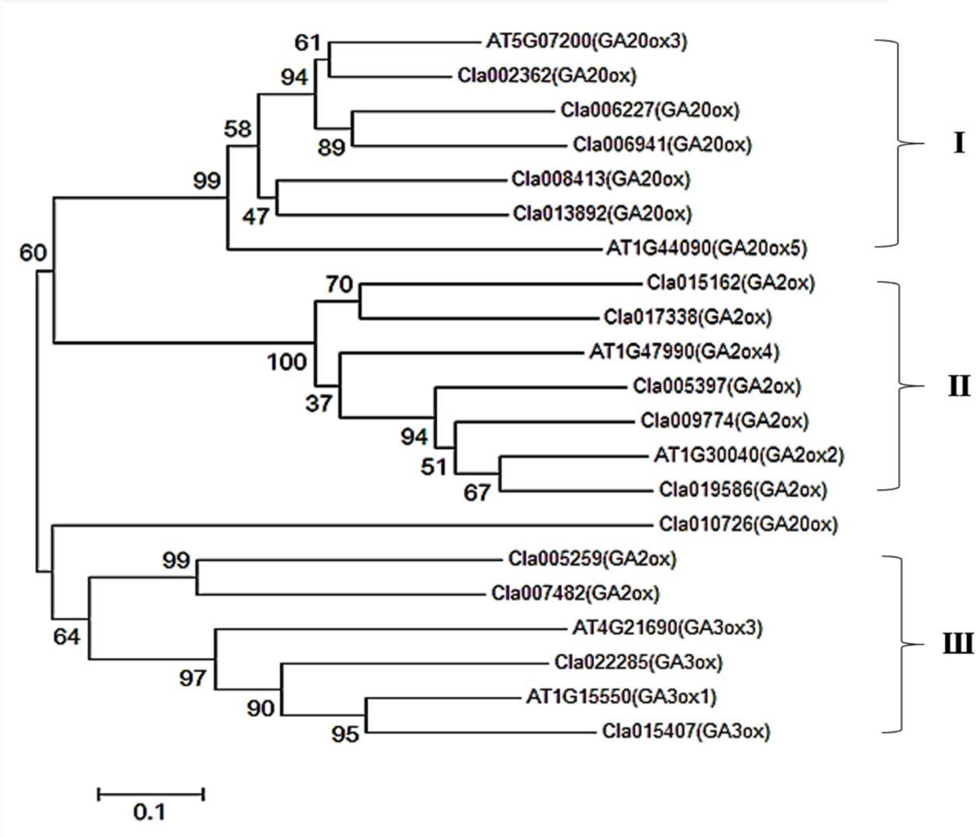

Fig. S1. Phylogenetic analysis of the GA20ox, GA3ox and GA2ox gene family in watermelon and those in Arabidopsis. Phylogenetic analysis was performed using MEGA 5 software with a bootstrap method and 1000 replications.

Legends of supplementary tables

Table S1. Δ(SNP-index) values for the region on chromosome 9 (0.72–3.93 Mb) .

Table S2. Δ(InDel-index) values for the region on chromosome 9 (0.80–3.43 Mb).

Table S3. SSR markers used for fine mapping and the obtained recombinant of these markers.

Table S4. Predicted genes and their annotation in the 541-kb region.
